# Development of High-Performance Composite Cementitious Materials for Offshore Engineering Applications

**DOI:** 10.3390/ma18143324

**Published:** 2025-07-15

**Authors:** Risheng Wang, Hongrui Wu, Zengwu Liu, Hanyu Wang, Yongzhuang Zhang

**Affiliations:** School of Transportation and Civil Engineering, Shandong Jiaotong University, Changqing District, Jinan 250357, China; hongruiw0925@163.com (H.W.); 230044@sdjtu.edu.cn (Z.L.); 15588714481@163.com (H.W.); 18369249197@163.com (Y.Z.)

**Keywords:** high-performance composite cementitious materials, composite cement mortar, mechanical properties, durability performance, microscopic analysis

## Abstract

This study focuses on the development of high-performance composite cementitious materials for offshore engineering applications, addressing the critical challenges of durability, environmental degradation, and carbon emissions. By incorporating polycarboxylate superplasticizers (PCE) and combining fly ash (FA), ground granulated blast furnace slag (GGBS), and silica fume (SF) in various proportions, composite mortars were designed and evaluated. A series of laboratory tests were conducted to assess workability, mechanical properties, volume stability, and durability under simulated marine conditions. The results demonstrate that the optimized composite exhibits superior performance in terms of strength development, shrinkage control, and resistance to chloride penetration and freeze–thaw cycles. Microstructural analysis further reveals that the enhanced performance is attributed to the formation of additional calcium silicate hydrate (C–S–H) gel and a denser internal matrix resulting from secondary hydration. These findings suggest that the proposed material holds significant potential for enhancing the long-term durability and sustainability of marine infrastructure.

## 1. Introduction

The construction industry is currently facing the dual challenge of ensuring structural durability while achieving carbon emissions reduction and sustainable development goals. In 2023, global carbon dioxide emissions reached a historic high of 35.8 billion tons, with cement production accounting for approximately 7–8% of the total, making it one of the major sources of industrial CO_2_ emissions [1,2]. In particular, traditional cement-based materials used in coastal and marine engineering applications not only have a high carbon footprint but also face significant durability challenges. These structures are frequently subjected to harsh environmental conditions, including chloride ion ingress, freeze–thaw cycles, and wet–dry alternation throughout their service life [3]. However, studies have shown that existing cementitious repair mortars commonly suffer from high shrinkage, poor resistance to chloride ion penetration, and insufficient long-term service performance, making them inadequate to meet practical engineering demands. Therefore, the development of novel cementitious materials that combine low carbon emissions with high durability is urgent. One promising strategy involves partially replacing Portland cement with supplementary cementitious materials (SCMs) to reduce the carbon footprint throughout the material’s lifecycle. Industrial by-products, such as fly ash (FA), ground granulated blast furnace slag (GGBS), and silica fume (SF), exhibit pozzolanic or latent hydraulic activity. During hydration, these SCMs react with calcium hydroxide (CH) to form additional calcium silicate hydrate (C–S–H) gel, improving the microstructure and enhancing long-term mechanical properties [4,5,6].

FA particles are spherical and help to fill pores in the cement paste, resulting in a denser structure and reducing the formation of cracks and weak zones [7]. Due to its relatively low reactivity, replacing cement with FA often lowers early-age strength but gradually enhances strength at later ages [8]. Zhang et al. [6] observed that the 28-day compressive, flexural, and axial compressive strengths of concrete initially increased and then decreased with rising FA content. The incorporation of FA also significantly promoted strength development at later stages. Balakrishnan [8] reported that fly ash plastering mortar can protect building interiors from chloride-induced corrosion. GGBS, a steel industry by-product, chemically binds chloride ions by forming Friedel’s salt, thereby reducing free chloride ion concentration. Specimens with 45% GGBS showed excellent durability, with permeability and water absorption improved by 17% and 18%, respectively, compared to reference specimens [9]. The active Al_2_O_3_ in GGBS significantly enhances the chloride-binding capacity of cement-based materials by participating in the hydration reaction. When used in combination with ordinary Portland cement (OPC), it notably improves the mechanical properties and chloride ion permeability resistance of the OPC–GGBS composite system [10]. Zhang et al. [11] demonstrated that slag micropowder improves workability and effectively enhances the later-age compressive strength and chloride-binding capacity of cement paste, with optimal performance at a 30% replacement level. Duan [12] showed that replacing cement with ground granulated blast furnace slag and metakaolin positively affects concrete’s pore structure and interfacial transition zone. The silica particles in SF are ultrafine and possess a larger specific surface area than cement particles. While cement particles tend to reduce workability by binding with the concrete or mortar matrix, the incorporation of SF effectively decreases the porosity of the mortar, forming a denser microstructure that enhances both durability and strength [13,14].

However, studies have shown that the performance improvement from using a single mineral admixture is limited; for example, fly ash exhibits low early-age reactivity, while high dosages of silica fume tend to deteriorate workability. Tong [15] found that replacing 40% of cement with metakaolin, calcined clay, fly ash, and slag improves early flexural and compressive strengths of cement mortar. Jiang et al. [16] compared chloride concentrations and diffusion coefficients under various mix proportions and concluded that binary blended cementitious materials have better resistance to chloride penetration than single-blended ones. Ahmad [17] suggested that the early compressive strength reduction in natural pozzolan-based concrete can be compensated by adding SF, which significantly improves durability without markedly increasing shrinkage. Anwar and Wang et al. [18,19] found that the use of by-product materials, such as FA and SF, in multicomponent cementitious systems with OPC improves concrete performance, notably enhancing its resistance to chloride ion penetration. Thomas et al. [3] demonstrated that cementitious materials with high slag content and low water-to-binder ratios reduce chloride diffusion rates. Low water-to-binder ratios also lower capillary porosity, decreasing permeability to water and other liquids [20]. The use of superplasticizers further optimizes cement mortar performance.

Despite significant advances in the development of cementitious materials for marine engineering, current commercial composite systems often prioritize individual properties, such as early strength or impermeability, without achieving a balanced enhancement across multiple performance indicators such as workability, mechanical strength, and durability. While SCMs, such as FA, GGBS, and SF, have been used to enhance concrete properties, the synergistic effects of these materials in combination with advanced chemical admixtures, such as polycarboxylate superplasticizers (PCEs), have not been adequately explored. This study addresses these gaps by developing a novel composite cementitious material that incorporates PCE and a ternary blend of FA, GGBS, and SF, optimized to improve both short-term and long-term performance under harsh marine conditions. The proposed material not only enhances mechanical properties and durability but also contributes to reduced carbon emissions, providing a more sustainable solution for marine infrastructure.

**Figure 1:**
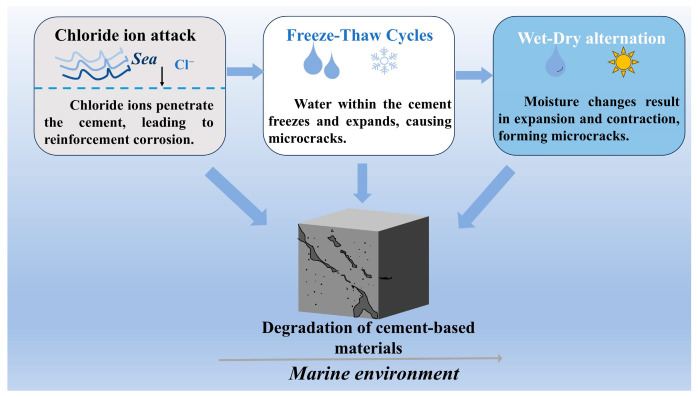
Degradation of cement-based materials in marine environments.

To better illustrate the degradation mechanisms of cementitious materials in marine environments, we have included a diagram that visualizes the key environmental factors contributing to the deterioration process. As shown in Figure 1, chloride ion attack, freeze–thaw cycles, and wet–dry alternation play critical roles in the degradation of cement-based materials, leading to structural deterioration. This diagram helps to clarify the challenges faced by cementitious materials exposed to harsh marine conditions and provides context for the subsequent experimental results.

## 2. Materials and Methods

### 2.1. Raw Materials

The cement used in this study was P·O 42.5 ordinary Portland cement produced by Shandong Cement Co., Ltd. (Jinan, China), with a specific surface area of 388 cm^2^/g. The FA was Class II fly ash supplied by Shanxi Longhui Building Materials Co., Ltd. (Datong, China). The GGBS was S95-grade slag powder provided by Longze Water Purification Materials Co., Ltd. (Gongyi City, China), with a specific surface area of 429 m^2^/kg. The SF was obtained from Yuanheng Water Purification Materials Factory (Gongyi City, China). Standard sand was used as the fine aggregate and tap water from the laboratory was used for mixing. The main chemical compositions of the raw materials are listed in Table 1.

The experiment employed two types of polycarboxylate-based high-performance superplasticizers, namely Solid Polycarboxylate Superplasticizer (PCE–S), produced by Tianjin Weihe Technology Development Co., Ltd. (Tianjin, China), and Liquid Polycarboxylate Superplasticizer (PCE–W), produced by Jiangsu Subote New Materials Co., Ltd. (Nanjing, China). The performance specifications of both types are presented in Table 2.

### 2.2. Specimen Preparation

**Figure 2:**
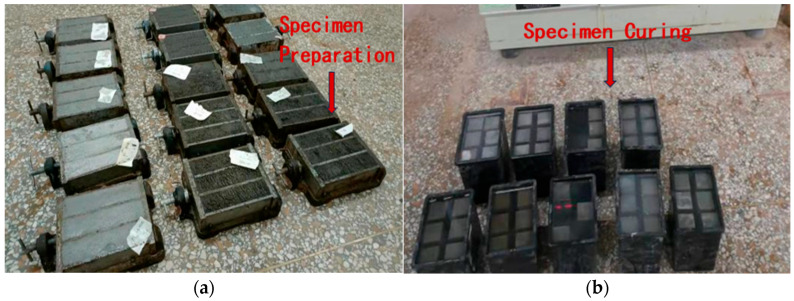
Preparation of cement mortar specimens: (**a**) preparation of cement mortar specimens; and (**b**) curing of cement mortar specimens.

Based on the designed mix proportions, the specified amounts of cement, mineral admixtures, standard sand, and powdered superplasticizer were precisely weighed and dry-mixed. Mixing water was then added and the mortar was blended using a cement mortar mixer in accordance with the JTG 3420-2020 [21] and the GB/T 17671-2021 [22]. Given that high-dosage superplasticizers may not fully exert their dispersing effect under low water-to-binder ratio conditions, the mixing time was appropriately extended during the experiment. The mixing was considered complete when the mixture was fully dispersed and homogeneous, typically after 1.5 min of high-speed agitation. The cement mortar was cast using a vibrating table and standard triple-section molds with dimensions of 40 mm × 40 mm × 160 mm, as shown in Figure 2a. After casting, the specimens were cured in the molds for 24 h before demolding. For mixes exhibiting delayed setting behavior, the curing period in the molds was extended to 48 h. Specimens were cured both before and after demolding, as illustrated in Figure 2b.

### 2.3. Test Methods

#### 2.3.1. Flowability Test

**Figure 3:**
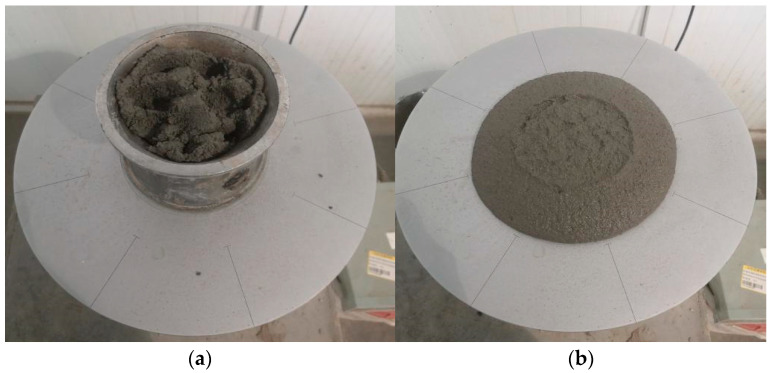
Flowability test of cement mortar: (**a**) filling and rodding of cement mortar; and (**b**) spreading and measurement of cement mortar.

As one of the key indicators of workability for both ordinary and composite cement mortars, flowability was evaluated in this study following the GB/T 2419-2005 [23]. A cement mortar flow table (commonly known as a “flow table”) was used to measure the flowability, as illustrated in Figure 3a,b. According to GB/T 2419-2005, the standard design value for the flowability of cement mortar is 180 mm. For cement mortar incorporating superplasticizers or composite cement mortar, the target flowability is adjusted to ensure proper molding without bleeding or insufficient cohesion, generally ranging between 150 and 180 mm.

#### 2.3.2. Flexural and Compressive Strength Tests

**Figure 4:**
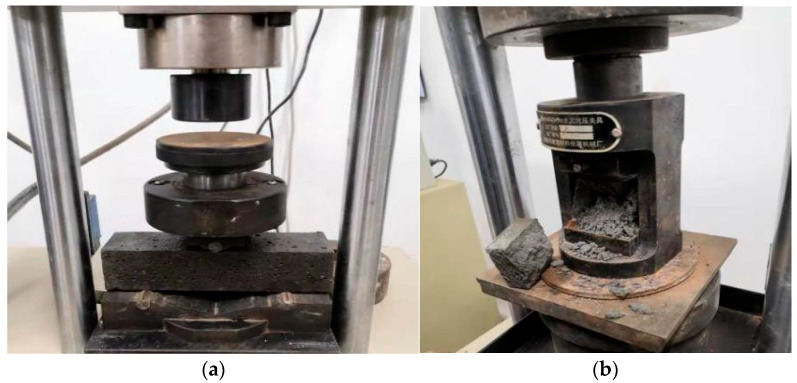
Cement mortar strength tests: (**a**) flexural strength test; and (**b**) compressive strength test.

According to the provisions of GB/T 17671-2021, the mechanical properties of the flexural and compressive strength for the prepared composite cement mortar specimens were tested using a YAW-3000 testing machine with Grade I accuracy (Xinguang Testing Instrument Co., Ltd., Cangzhou, China), as shown in Figure 4a,b. The specimen dimensions are 40 mm × 40 mm × 160 mm. For each group, 3 flexural strength values and 6 compressive strength values were obtained. During data processing, if any flexural or compressive strength value exceeded the average by more than ±10%, it was excluded and the average was recalculated. For compressive strength, if, after exclusion, any of the remaining 5 values deviated from the average by more than ±10%, they were considered invalid data.

#### 2.3.3. Tensile Strength Test

**Figure 5:**
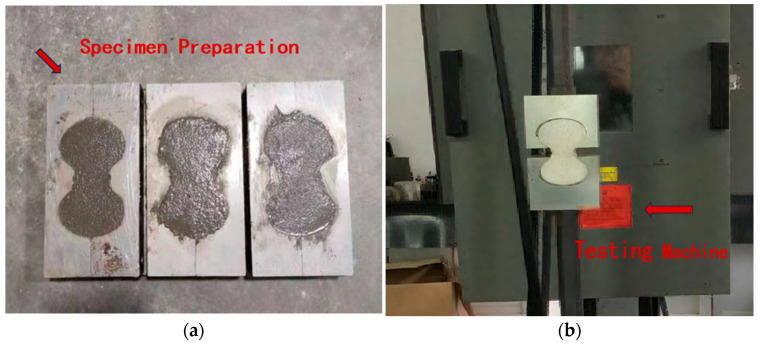
Tensile strength test: (**a**) formation of the tensile strength specimen; and (**b**) strength measurement using the testing machine.

According to the requirements specified in the JTS/T 232-2019 [24] and the JTS/T 311-2023 [25], the tensile strength of the repair mortar was determined. Three ‘8’-shaped specimens were prepared per group, as shown in Figure 5a. The specimens were demolded after 24 h of casting and then cured in a constant temperature and humidity chamber at 20 ± 2 °C and 65 ± 5% relative humidity for up to 14 days. Tensile strength was measured using an MTS-810 material dynamic testing system (MTS Systems Corporation, Eden Prairie, MN, USA), as shown in Figure 5b. The tensile strength was calculated according to Equation (1) and the average of three measurements was taken as the final result:

(1) $$f_{t}=\frac{P}{S}$$

where

$f_{t}$—Tensile strength, in MPa;

P—Failure load, in N;

S—Failure cross-sectional area of the figure eight-shaped specimen, in mm^2^.

#### 2.3.4. Bond Strength Test

**Figure 6:**
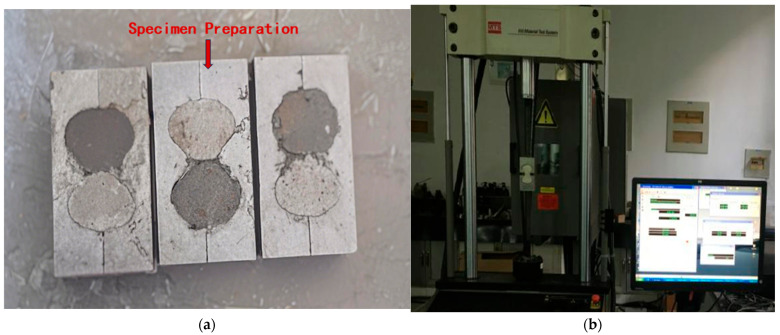
Bond strength test: (**a**) bond strength specimen molding; and (**b**) bond strength measurement.

“8”-shaped specimens were prepared, following the tensile test requirements. During molding, a metal plate was inserted at the center. After demolding, the two halves were separated and cured for 28 days. Half of the specimens were then placed back into the “8”-shaped mold, and an interface bonding agent was applied to the fracture surface. Freshly mixed repair mortar was poured into the blank half of the mold to remold and form new-to-old cement mortar bonding specimens. The bonded specimens were cured under the same conditions for 28 days. The bond tensile strength was then measured using the MTS-810 material testing system. The average of three measurements was taken as the final result, as shown in Figure 6a,b. Bond strength was calculated according to Equation (1) and was required to be no less than the tensile strength standard of the original concrete structure.

#### 2.3.5. Dry Shrinkage Test

**Figure 7:**
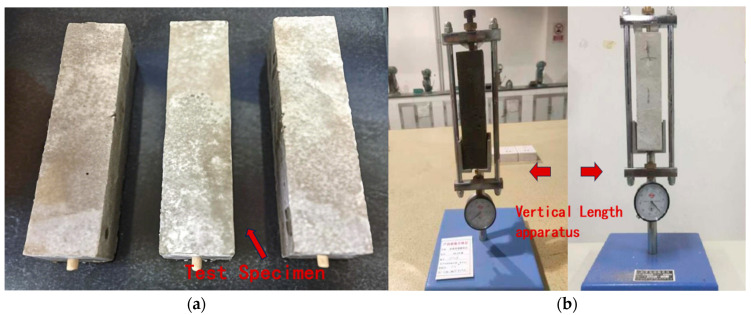
Drying shrinkage test: (**a**) dry shrinkage test specimen; and (**b**) length measurement using vertical shrinkage apparatus.

According to the requirements of JTS/T 232-2019 and JTS/T 311-2023, mortar specimens were prepared using molds measuring 40 mm × 40 mm × 160 mm. Each mold features a hemispherical groove with a radius of 2.5 mm at the center of both end forms. Three specimens were prepared per group, as shown in Figure 7a. The molding process was carried out in two layers, with 10 fillings and roddings per layer. After molding, the specimens were placed in a curing box at 20 ± 2 °C with a relative humidity of no less than 80%. After 48 h, the molds were removed and the specimens are then placed in water at 20 ± 2 °C for an additional 5 days of water curing. Afterward, the specimens are removed, dried, and their reference length was measured using a vertical shrinkage device, as shown in Figure 7b. The specimens were then placed in a curing chamber at 20 ± 2 °C and a relative humidity of 65 ± 5% until the designated curing age, after which their lengths were measured. Dry shrinkage was calculated according to Equation (2), with an accuracy of 1 × 10^−6^. The average value of three specimens per group was taken as the dry shrinkage rate at the corresponding curing age, as follows:

(2) $$\varepsilon_{t}=\frac{L_{t}\;-\;L_{0}}{L_{0\;}-\;2\Delta }$$

$\varepsilon_{t}$—Dry shrinkage at a given curing age, in με;
$L_{0}$—Initial reference length of the specimen, in mm;
$L_{t}$—Length of the specimen at the corresponding curing age, in mm;
Δ—Length of the metal gauge head, in mm.

#### 2.3.6. Freeze–Thaw Cycling Test

**Figure 8:**
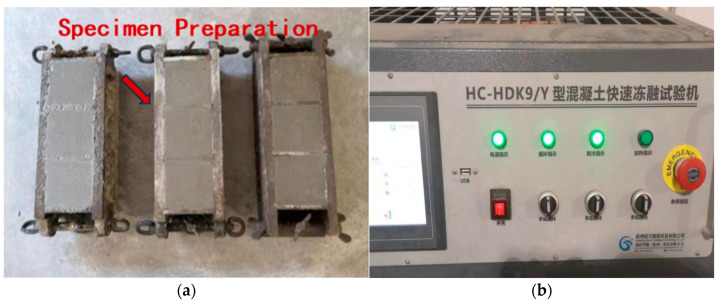
Freeze–thaw cycling test: (**a**) specimen molding; and (**b**) fully automated rapid freeze–thaw cycling.

The frost resistance performance of the specimens was evaluated in accordance with the JTS/T 236-2019 [26]. Cube specimens with dimensions of 70.7 mm × 70.7 mm × 70.7 mm were used. Two sets of specimens were prepared for each mix composition: one set (three specimens) was reserved as a control group for compressive strength measurement, as shown in Figure 8a. The HC-HDK9/Y rapid freeze–thaw testing machine (Hangzhou Guanli Intelligent Technology Co., Ltd., Hangzhou, China). was used to perform the test, as shown in Figure 8b. Specimens that had reached the designated curing age were removed from the curing chamber for visual inspection. The freeze–thaw medium was seawater. Before testing, the specimens were air-dried for two days and then immersed in seawater for another two days. During the freeze–thaw cycling process, the freezing duration should be approximately 4 h, and the thawing time in water should not be less than 4 h. The temperature ranges for freezing and thawing are −20 °C to −15 °C and 15 °C to 20 °C, respectively. The ambient laboratory temperature during the test should be maintained at 20 ± 2 °C.

#### 2.3.7. Chloride Ion Permeability Test

**Figure 9:**
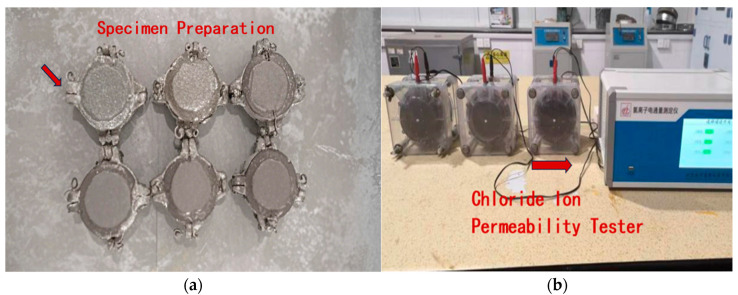
Chloride ion permeability test: (**a**) specimen molding; and (**b**) testing setup.

In accordance with the (JGJ/T 193-2009) [27], the electrical flux method was employed for the study. Cylindrical specimens with a height of 50 mm and a diameter of 100 mm were prepared, as shown in Figure 8a, with an error margin controlled within ±1 mm. The test measures the charge passed over a specific period to characterize the ability of concrete to resist chloride ion penetration. The electrical flux of composite cementitious mortar specimens, cured for the required age, was measured. The average value of three specimens per group was taken as the electrical flux value. The test setup is shown in Figure 9b.

#### 2.3.8. Permeability Resistance Test

**Figure 10:**
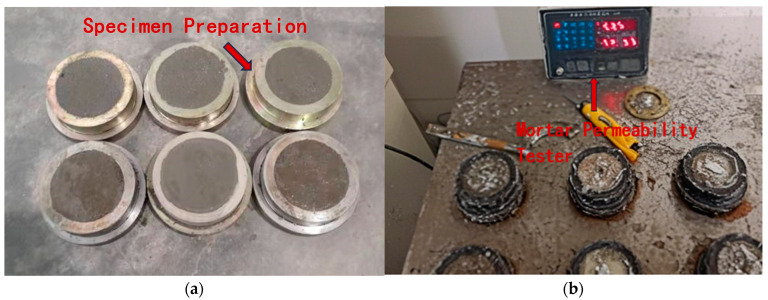
Permeability resistance test: (**a**) specimen molding; and (**b**) mortar permeability test using the permeability apparatus.

The permeability performance of the specimens was evaluated according to the specifications in JTS/T 236-2019. The conical mold used for the permeability test has a height of 30 mm, with the upper and lower diameters being 70 mm and 80 mm, respectively. The test molds were placed on the vibration table for compaction and the surface was leveled to form the specimens. The equipment used for the test was a mortar permeability tester. For each mix of cementitious mortar, six specimens were prepared per group, as shown in Figure 10a. After molding, the specimens were left to stand at room temperature (20 ± 5 °C) for 24 ± 2 h before demolding and then cured until the specified age. The specimens were subsequently subjected to permeability testing. According to the standard procedure, water pressure was applied; the test was terminated when surface seepage was observed in three out of six specimens. When the water pressure was held constant, the permeability performance was evaluated based on the maximum pressure at which at least four out of six specimens remained free of seepage, as illustrated in Figure 10b.

The calculation of impermeability pressure is given by Equation (3):

*P* = *H* − 0.1 (3)

where:

*P*—Impermeability pressure of cement mortar, in MPa;
*H*—Water pressure at the cessation of the permeability test, in MPa.

### 2.4. Mix Design and Preliminary Performance Testing

In both Plan 1 and Plan 2, PCE-S and PCE-W were selected as the sole additives, respectively. The dosage of superplasticizer, water content, and water-to-binder ratio were adjusted as three factors. Flowability, as well as 28-day flexural and compressive strengths, was used as an evaluation indicator. Based on the workability and mechanical performance, the optimal mix design for the cement mortar was determined. Through orthogonal experimental design, the optimal mix for Plan 1 was identified as PCE-S1; the optimal mix for Plan 2 was identified as PCE-W1, as shown in Table 3.

This study investigates the mix design of composite cementitious mortar based on PCE-S1 and PCE-W1. FA and GGBS were used as mineral admixtures, replacing cement in a 1:3 FA:GGBS ratio. The total dosage of these mineral admixtures was varied at 10%, 20%, 30%, 40%, and 50% to evaluate their effects. Flowability, along with the 28-day flexural and compressive strengths, were used as evaluation criteria. Based on these findings, the optimal dosage combination was selected. The internal incorporation method was then applied, where SF was added at dosages of 2%, 5%, and 8%, while keeping the total dosage of the mineral admixtures constant. The ratio of the three mineral admixtures—FA:GGBS:SF = 1:3:X—was treated as the variable, with X taking values of 0.2, 0.6, and 1.0.

Mix Design Scheme 3 was developed based on the optimal mix design of Scheme 1 (PCE-S1). The detailed proportions are presented in Table 4.

As shown in Table 4, the flowability values of all mixtures range from 150 mm to 166 mm, which meet the design targets of the mix proportions.

For the composite cementitious mortar containing supplementary cementitious materials, considering that strength continues to increase beyond 28 days of curing, both the 28-day and 56-day strength values were used as evaluation indicators. PCE-S5 was identified as the optimal mix ratio, with the best total dosage of FA:GBSS = 1:3 at 40%. Based on this total dosage of mineral admixture, the mix design for composite cementitious mortar incorporating SF was developed. The ratios of the mineral admixture were set as 1:3:0.2, 1:3:0.6, and 1:3:1.0, with the corresponding test results shown in Table 5.

Based on the results in the table, the optimal mix design for this scheme is PCE-S8, with a total mineral admixture content of 40% and the proportions of the three mineral admixtures—FA, GGBS, and SF—set at 1:3:0.6.

Mix Design Scheme 4 was developed by incorporating mineral admixtures into the optimal mix of Scheme 2 (PCE-W1). The types and proportions of mineral admixtures used were identical to those in Scheme 3. The detailed mix proportions and corresponding results for the composite cement mortars are presented in Table 6.

From Table 6, PCE-W5 was selected as the optimal mix design, with the best total dosage of mineral admixture at 40% when the FA:GBSS ratio is 1:3. Based on this total dosage of mineral admixture, the mix design for the composite cementitious mortar incorporating internal SF was carried out. The ratios of the three mineral admixtures were varied as part of a single-factor experiment. The experimental results are presented in Table 7.

Similar to Scheme 3, the optimal mix design for this scheme, based on achieving appropriate flowability and maximizing mechanical strength, is PCE-W8. It uses a total mineral admixture dosage of 40%, with the mineral admixture ratio remaining at FA:GBSS:SF = 1:3:0.6.

## 3. Performance Study of High-Performance Composite Cementitious Mortar for Marine Engineering

### 3.1. Workability of Marine Engineering High-Performance Composite Cementitious Mortars

**Figure 11:**
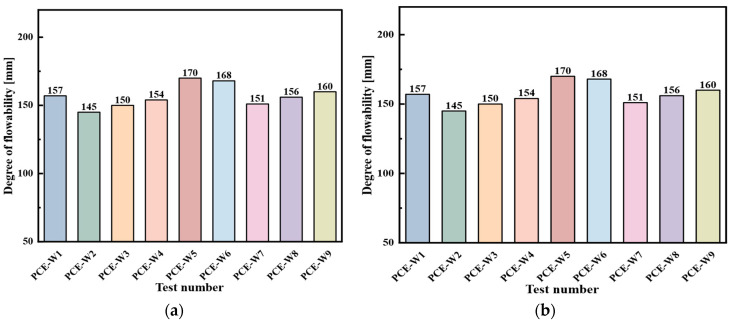
Flowability of composite cementitious mortar for Scheme 3 and Scheme 4: (**a**) Scheme 3; and (**b**) Scheme 4.

The flowability of composite cement mortars containing two types of polycarboxylate superplasticizers, PCE-S and PCE-W, was analyzed under varying types and dosages of mineral admixtures. Among these, PCE-S1 and PCE-W1 served as control specimens without any mineral admixture. The comparative results of all mix designs are presented in Figure 11.

From Figure 11, it can be seen that in both Scheme 3 and Scheme 4, when only FA and GGBS are used as mineral admixtures, the flowability increases with the dosage. However, when the dosage of the admixtures is ≤30%, the flowability values are lower than those of the two blank control groups. When the dosage exceeds 30%, the flowability gradually approaches or exceeds that of the blank control groups. When three types of mineral admixtures are used, with the total dosage kept constant, the flowability increases as the proportion of SF rises.

The results indicate that, except for PCE-S9—which contains a relatively high proportion of silica fume—and PCE-W2, where the mineral admixture dosage is too low, the flowability values of the remaining mixes fall within the target design range. Using flowability as the evaluation criterion, the incorporation of a small amount of mineral admixtures adversely affects the workability of the composite cementitious mortar. Significant improvement in flowability is observed only when the dosage exceeds 30%.

### 3.2. Mechanical Properties of Marine Engineering High-Performance Composite Cementitious Mortars

#### 3.2.1. Effect of Mineral Admixture Type and Dosage on Flexural and Compressive Strength

**Figure 12:**
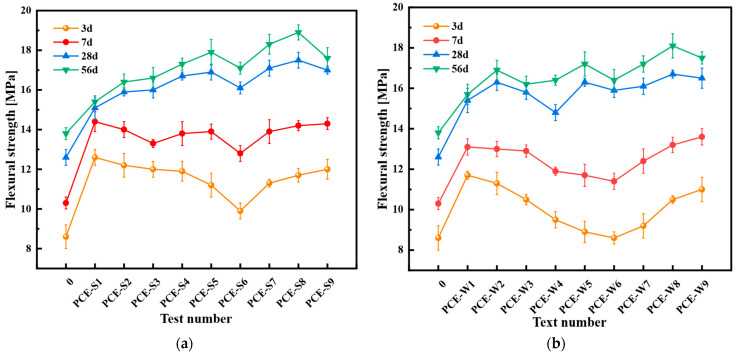
Flexural strength of composite cementitious mortar for Scheme 3 and Scheme 4: (**a**) Scheme 3; and (**b**) Scheme 4. Note: Error bars indicate the standard deviation of three replicate measurements.

**Figure 13:**
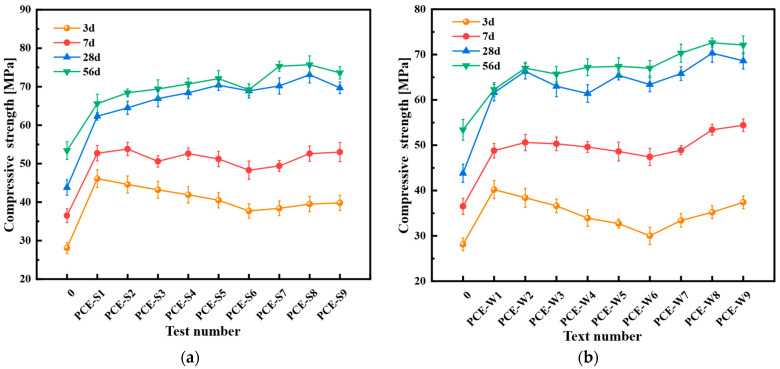
Compressive strength of composite cementitious mortar for Scheme 3 and Scheme 4: (**a**) Scheme 3; and (**b**) Scheme 4. Note: Error bars indicate the standard deviation of six replicate measurements.

This study aimed to investigate the effect of the type and dosage of mineral admixtures on the flexural and compressive strengths. PCE-S1 and PCE-W1 served as the blank control specimens. Test No. 0 represents the ordinary cement mortar mix without any admixtures or mineral admixtures, serving as the control group. The results are presented in Figure 12 and Figure 13.

As shown in Figure 12, the flexural strength of all mix designs is higher than that of the plain cement mortar with test number 0. With the increase in curing age, the flexural strength of Schemes 3 and 4 significantly improves. For most mix designs, the flexural strength of the composite cementitious mortar at 3 days and 7 days is lower than that of the blank specimens, PCE-S1 and PCE-W1. However, when the content of the mineral admixtures is 40%, with a larger proportion of SF, the flexural strength of the cementitious mortar becomes comparable to, or higher than, that of the blank specimens, with the PCE-W mix design showing a more pronounced effect.

Compared to the blank specimens PCE-S1 and PCE-W1, the flexural strength of all mix designs incorporating mineral admixtures is greater than both. Specifically, PCE-S5 shows an increase of 11.9% at 28 days and 16.2% at 56 days compared to PCE-S1; PCE-S8 exhibits increases of 15.9% and 22.7%, respectively. When compared to PCE-W1, PCE-W5 shows an increase of 6.0% and 9.5%, while PCE-W8 shows increases of 9.2% and 15.3% at 28 and 56 days, respectively. In terms of flexural strength, a trend gradually becomes apparent after 28 days: as the content of the mineral admixtures and the proportion of SF increase, the flexural strength first increases and then decreases. PCE-S8 shows a flexural strength of 17.5 MPa at 28 days and 18.9 MPa at 56 days; PCE-W8 exhibits strengths of 16.7 MPa and 18.1 MPa at 28 and 56 days, respectively, representing the highest strength values among all mix designs. Both PCE-S8 and PCE-W8 have a mineral admixture content of 40%, with a ratio of the three mineral admixtures being 1:3:0.6.

As shown in Figure 13, the compressive strength of the cementitious mortar in Schemes 3 and 4 exceeds that of the blank specimen starting at 7 days, especially for the mix designs incorporating PCE-W, which exhibit higher strength values than the blank specimen PCE-W1 without any mineral admixtures. To maintain consistency under the same conditions, the compressive strength analysis refers to the values at 28 days and 56 days. The compressive strength of PCE-S5 increased by 13.0% at 28 days and 9.9% at 56 days compared to PCE-S1, while PCE-S8 showed increases of 17.3% and 15.4%, respectively. Compared to PCE-W1, PCE-W5 exhibited increases of 6.2% and 8.2%, and PCE-W8 showed increases of 14.1% and 16.5%. The 28-day and 56-day compressive strength of PCE-S8 and PCE-W8 were the highest among all mix designs using similar superplasticizers.

The composite cement mortar incorporating mineral admixtures exhibits relatively low early-age strength, with compressive strength significantly increasing by 7 days and flexural strength showing marked improvement at 28 days. As the total mineral admixture content and the proportion of silica fume increase, these strength values initially rise and then decline, with this trend becoming more pronounced beyond 28 days. The study demonstrates that adding silica fume alongside fly ash and slag greatly enhances both flexural and compressive strength. To achieve optimal performance, the mineral admixture content should be maintained within a suitable range. Specifically, a total mineral admixture content of 40%, with a ratio of FA:GGBS:SF = 1:3:0.6, is recommended.

#### 3.2.2. The Effect of Different Mix Designs on Tensile Strength

**Figure 14:**
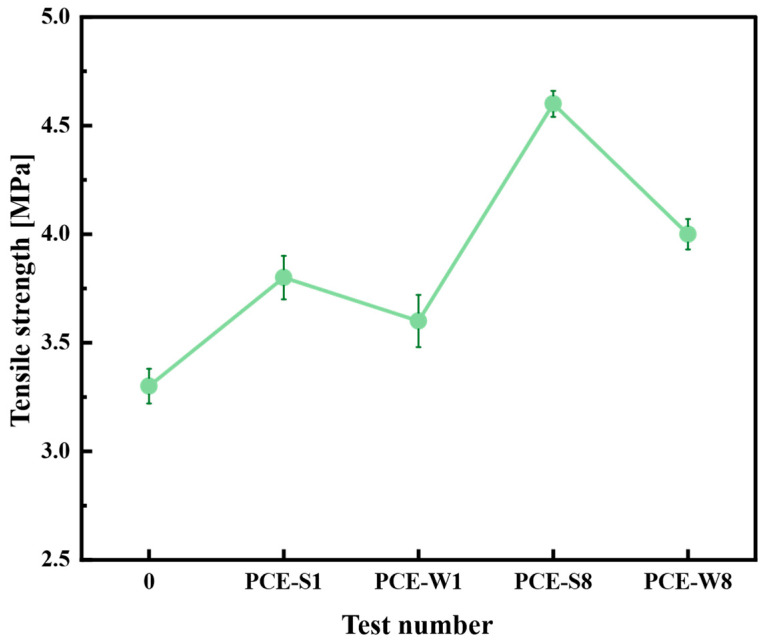
Tensile strength of different mix designs. Note: Error bars indicate the standard deviation of three replicate measurements.

Based on the previous experimental design of composite cementitious mortar mix ratios and the analysis of key performance indicators, such as flowability, flexural strength, and compressive strength, the optimal mix ratios were selected from Schemes 1, 2, 3, and 4. These were PCE-S1, PCE-W1, PCE-S8, and PCE-W8, respectively. The tests were conducted in accordance with the relevant requirements of JTS/T 232-2019 and JTS/T 311-2023 for the molding and curing of composite cementitious mortar with the four different mix designs mentioned above. The specimens were cured until the age of 14 days and their corresponding tensile strengths were measured. Three specimens were prepared for each mix design, with the test results being presented as the arithmetic average, accurate to 0.1 MPa. Using the ordinary Portland cement mortar with test number 0 as the blank specimen for comparison, the maximum tensile force for each mix design was obtained. The tensile strength was calculated by dividing the maximum tensile force by the cross-sectional area. The results of the different tensile strengths are shown in Figure 14.

As shown in Figure 14, the tensile strength of composite cementitious mortar is significantly improved with the incorporation of superplasticizers and mineral admixtures. The tensile strength of the blank sample (Test No. 0) at 14 days was 3.3 MPa. In comparison, the tensile strength of the mortar with only superplasticizers, PCE-S1 and PCE-W1, increased by 15.2% and 9.1%, respectively. This demonstrates that adding an appropriate amount of superplasticizer can significantly enhance the tensile strength of cement mortar, with solid superplasticizer performing better than the liquid type. Moreover, when mineral admixtures are incorporated at 40%, as in PCE-S8 and PCE-W8, the tensile strength increased by 21.0% and 11.1%, respectively, compared to PCE-S1 and PCE-W1 without mineral admixtures. Therefore, for engineering applications, it is recommended to prioritize the combined use of solid superplasticizer and mineral admixtures.

#### 3.2.3. The Effect of Different Mix Designs on Bond Strength

**Figure 15:**
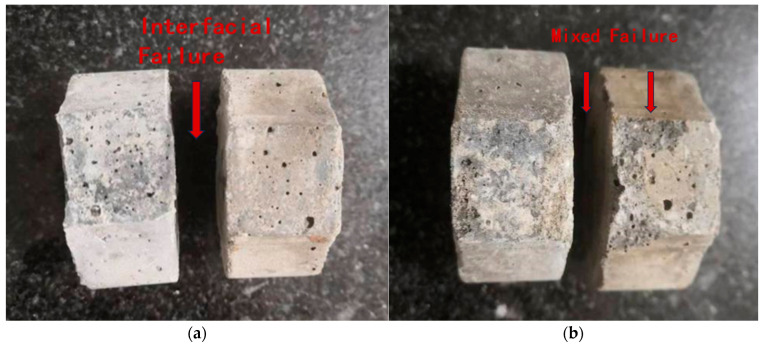
Bond failure characteristics of existing and fresh cement mortar specimens: (**a**) interface failure; and (**b**) mixed failure.

In accordance with JTS/T 232-2019, composite cementitious mortars with different mix designs, including PCE-S1, PCE-W1, PCE-S8, and PCE-W8, were prepared, and the specimens were molded and cured. After curing for 28 days, the bond tensile strength was measured. In this experiment, the interface between the existing and fresh cement mortar is located at the point of minimum cross-sectional area. The tensile strength of both the composite cementitious mortar and ordinary cement mortar is relatively high. As a result, the failure modes observed in the bond strength test include interface failure as well as combined failure, involving both the internal structure of the existing and fresh cement mortars and the interface. These failure modes are illustrated in Figure 15a,b.

**Figure 16:**
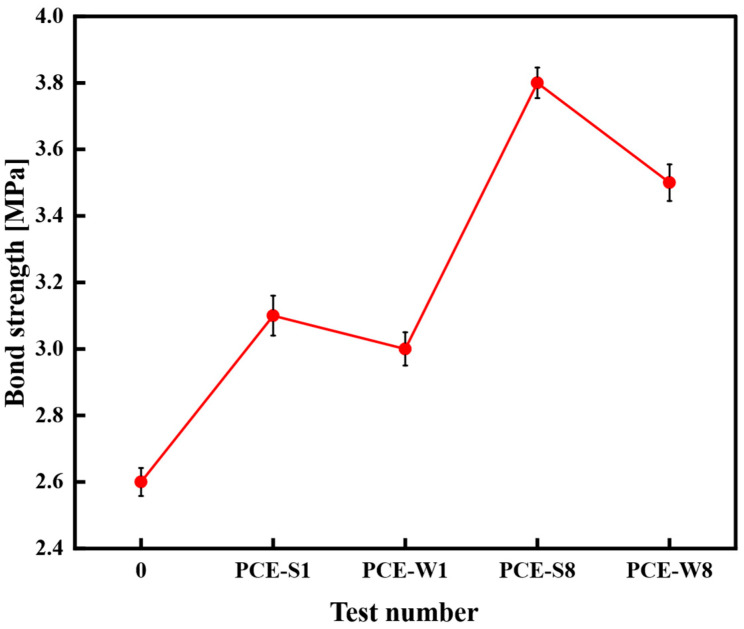
Bond strength of different mix designs.

The average of three measured values was used as the test result, as shown in Figure 16.

From Figure 16, it can be seen that the bond strength of composite cementitious mortars containing superplasticizers is higher than that of the blank specimen (Test No. 0), which has a bond strength of 2.6 MPa. Among them, the bond strength of the PCE-S1 composite cementitious mortar is higher than that of PCE-W1. Furthermore, composite cementitious mortars with a 40% mineral admixture, namely PCE-S8 and PCE-W8, show an improvement in bond strength compared to PCE-S1 and PCE-W1, with increases of 22.6% and 16.7%, respectively. PCE-S8 exhibits the largest increase, with a bond strength of 3.8 MPa, which represents a 46.2% improvement over the blank specimen (Test No. 0), while still meeting the performance standards.

Both superplasticizers and mineral admixtures can significantly improve the bond strength of composite cementitious mortars, with solid superplasticizers having a more pronounced effect on performance enhancement.

### 3.3. Volume Stability of Marine Engineering High-Performance Composite Cementitious Mortars

**Figure 17:**
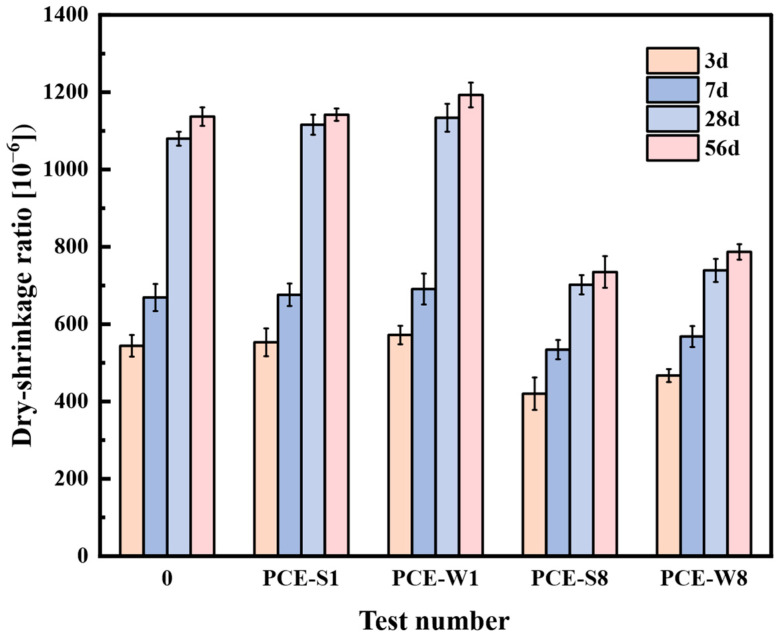
Drying shrinkage rates of different mix designs at various ages.

The experiment used a vertical shrinkage apparatus to measure the drying shrinkage of four different cement mortar mix designs at 3, 7, 28, and 56 days, with the blank specimen (Test No. 0) serving as the control. The drying shrinkage rates for each mix design were calculated using Equation (1); the results are presented in Figure 17.

As shown in Figure 17, with the increase in curing age, the drying shrinkage rates of all mix designs gradually increase. However, after 28 days, the rate increase in drying shrinkage for all mix designs decreases, indicating that the axial length deformation has stabilized. Therefore, the study of the drying shrinkage behavior of composite cementitious mortars and their volume stability should focus on the period before 28 days.

The drying shrinkage rates of the composite cementitious mortars with only the superplasticizers, PCE-S1 and PCE-W1, were consistently higher than that of the blank specimen with test number 0, which contained no superplasticizer. The drying shrinkage rate of PCE-W1 was slightly higher than that of PCE-S1 at different ages.

The drying shrinkage rates of PCE-S8 and PCE-W8 were not only lower than those of the control groups PCE-S1 and PCE-W1, which contain no mineral admixtures, but also lower than the blank specimen with test number 0. Due to the higher proportion of GBSS, which exhibits pozzolanic effects, micro-aggregate effects, and micro-crystal nucleation effects, the early hydration water demand was lower than that of ordinary Portland cement, thereby reducing drying shrinkage. SF can improve the pore structure of cement-based materials, contributing to enhanced volume stability. Studies have shown that incorporating mineral admixtures helps to reduce the drying shrinkage rate of composite cementitious mortars, thereby improving their volume stability. The optimal mix design should be PCE-S8, which combines solid superplasticizers and mineral admixtures.

### 3.4. Durability of Marine Engineering High-Performance Composite Cementitious Mortars

#### 3.4.1. Experimental Study on Frost Resistance

**Figure 18:**
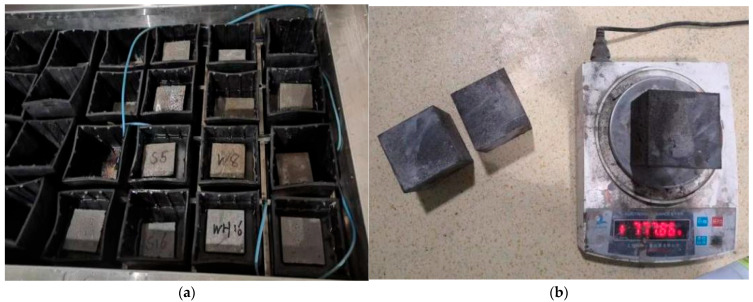
Freeze–thaw performance test of composite cementitious mortars: (**a**) freeze–thaw of specimen; and (**b**) mass measurement of specimen.

Four different mix designs of composite cementitious mortars (PCE-S1, PCE-W1, PCE-S8, and PCE-W8) were prepared, and the freeze–thaw resistance of specimens cured for 28 days was measured using a direct freeze–thaw method, as shown in Figure 18a. It is important to note that the test should be terminated if the following conditions occur during the freeze–thaw cycles: (1) the mass loss rate of the specimen exceeds 5%; (2) the compressive strength loss rate of the specimen exceeds 25%. If the mass loss rate of the freeze–thaw specimen is no greater than 5% and the compressive strength loss rate is no greater than 25%, the freeze–thaw resistance is considered qualified for this number of cycles; otherwise, it is considered unqualified. The measurements are shown in Figure 18b.

The test uses specimen 0 as the control. The number of freeze–thaw cycles, mass loss rate, and compressive strength loss rate of each mix design are shown in Table 8 and Table 9 below.

**Figure 19:**
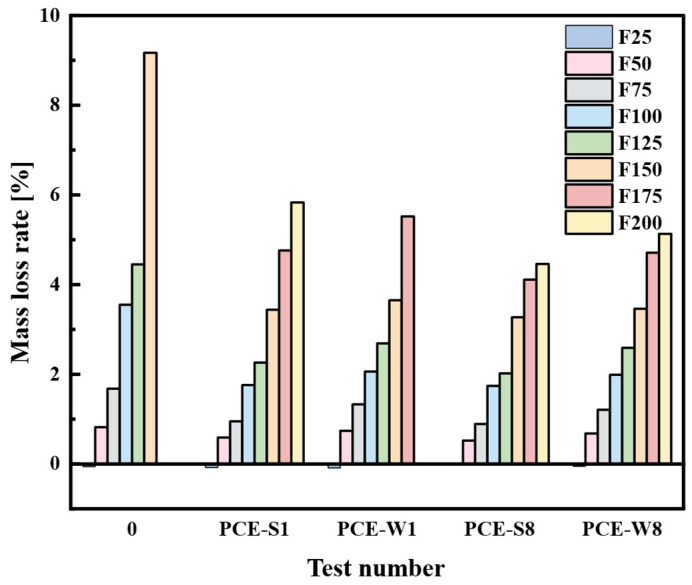
Mass loss rate of each mix design at different freeze–thaw cycles.

The relevant data in the table are plotted as a bar chart, as shown in Figure 19.

From Figure 19, it can be seen that after 25 freeze–thaw cycles (F25), the mass of all five groups of cement mortar specimens increased. This phenomenon may be attributed to the freeze–thaw-induced expansion pressure, which generates microcracks and enlarges existing capillary pores. As a result, the internal porosity increases, allowing for more water to be retained within the matrix during the thawing stage, thereby leading to a net gain in mass. After F50, the mass of all five groups of composite cement mortar specimens decreased. As the number of freeze–thaw cycles increased, different degrees of spalling or damage appeared on the specimen surfaces, with some specimens even fracturing. The control specimen (0) showed a mass loss of 9.17% at F150, exceeding the standard performance requirement of 5%, and was much higher than the mass loss observed in the PCE-S1 and PCE-W1 cement mortar specimens with superplasticizers under the same number of freeze–thaw cycles. This is because superplasticizers have an air-entraining effect, which significantly enhances the freeze–thaw resistance by improving the internal microstructure of the cement-based material. Compared with PCE-S1 and PCE-W1, the mass loss rates of PCE-S8 and PCE-W8 composite cement mortars are lower, and the effect on enhancing freeze–thaw resistance becomes significantly more pronounced after F175. At F175, PCE-W8 shows a 1.35% reduction in mass loss rate compared to PCE-W1; at F200, PCE-S8 shows a 1.37% reduction in the mass loss rate compared to PCE-S1. This indicates that the incorporation of a larger amount of mineral admixtures is effective in reducing mass loss during freeze–thaw cycles, with the effect becoming more significant as the number of cycles increases.

**Figure 20:**
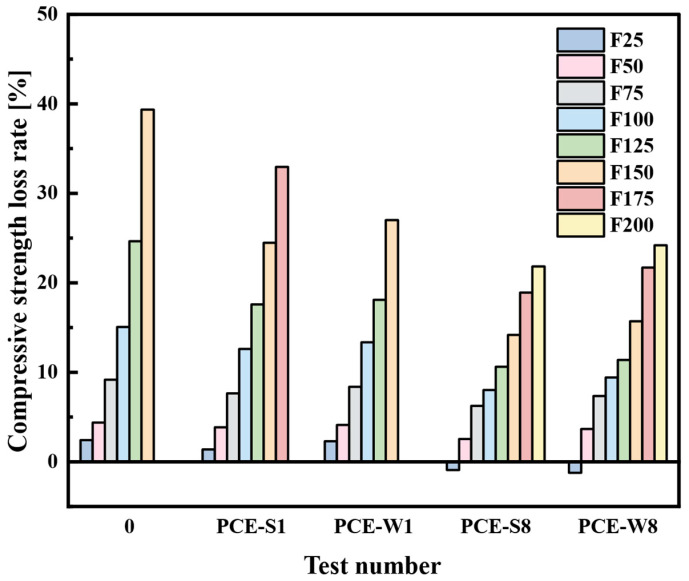
Mass loss rate of each mix design at different freeze–thaw cycle counts.

The data from the table are plotted in Figure 20.

From Figure 20, it can be seen that at F25, the compressive strength of PCE-S8 and PCE-W8 composite cementitious mortars both show an increasing trend, indicating that the cementitious mortars with a larger amount of mineral admixtures continue to undergo further hydration reactions after 28 days of curing, thereby increasing the mechanical strength. After F50, the strength of all five groups of specimens shows a decreasing trend. The decrease in strength of the blank specimen (Test No. 0) is significantly larger than the other mix designs. At F125, the compressive strength loss rate of the blank specimen reached 24.64%, which is close to the 25% limit specified in the performance standards. At F150, the strength decreased by 39.36%, which exceeds the limit by approximately 14.4%. The strength loss rates of PCE-S1 and PCE-W1 cement mortar are lower than that of the blank specimen (Test No. 0) at all freeze–thaw cycles. This is due to the air-entraining effect of the superplasticizer, which improves the internal micro-porous structure, thereby reducing strength loss. At F150, the strength loss rate of PCE-W1 exceeds 25%, while the strength loss rate of PCE-S1 is 24.47%. By F175, the strength loss rate of PCE-S1 reaches 32.95%. The improvement effect of the solid superplasticizer is superior to that of the liquid superplasticizer. Compared to PCE-S1 and PCE-W1 cement mortars, PCE-S8 and PCE-W8 exhibit significantly lower compressive strength loss. At F200, the strength loss rates of PCE-S8 and PCE-W8 are 21.83% and 24.20%, respectively, both of which do not exceed the freeze–thaw performance standard requirements. This indicates that mineral admixtures play an active role in improving the micro-porous structure of cement-based materials, which helps reduce mechanical strength loss. The incorporation of both water-reducing agents and mineral admixtures has a positive effect on improving the freeze–thaw resistance of cement mortars. The PCE-S8, with the combined addition of solid water-reducing agents and a relatively large amount of mineral admixtures, shows the best freeze–thaw performance, meeting the requirement of no fewer than 200 cycles in freeze–thaw tests under marine environmental conditions.

#### 3.4.2. Experimental Study on Chloride Ion Penetration Resistance

**Figure 21:**
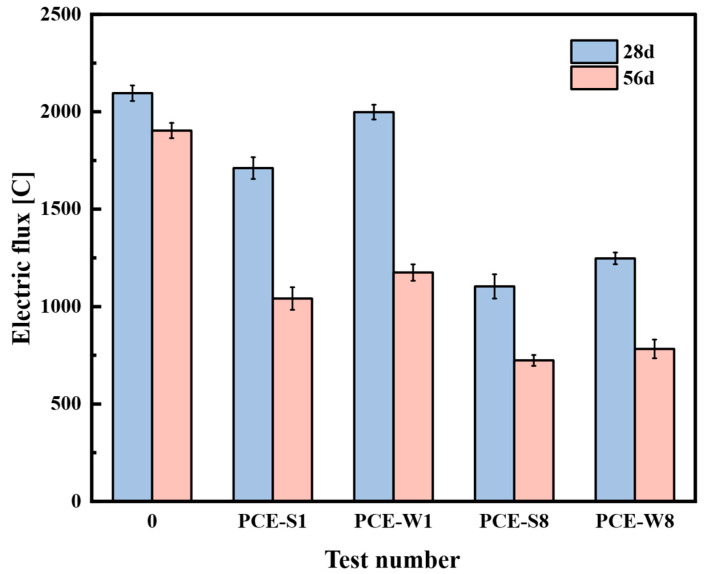
Chloride ion electrical flux of each mix design.

According to the specifications of JGJ/T 193-2009, the electric flux of composite cementitious mortars—PCE-S1, PCE-W1, PCE-S8, and PCE-W8—was measured at curing ages of 28 and 56 days, using the blank specimen (Test No. 0) as the control group to evaluate chloride ion permeability resistance. The current at both ends of each specimen was recorded every 30 min, and the total electric flux over a 6 h period was calculated. The average value of three specimens from each group was used to characterize the chloride ion penetration resistance. The test results are presented in Figure 21.

As shown in Figure 21, whether using superplasticizer alone or in combination with mineral admixtures, the electrical flux values of the composite cementitious mortar are significantly lower than that of the blank specimen, indicating a substantial improvement in resistance to chloride ion penetration. Notably, the performance of the composite cementitious mortar incorporating both superplasticizer and mineral admixtures is more pronounced. This can be attributed to the fact that the addition of fly ash promotes secondary hydration of cement, generating calcium silicate hydrate (C-S-H) and calcium sulfoaluminate hydrate (CAH). Additionally, the hydration products of GBSS act as filling materials at the interface with the cementitious matrix, while the microfilling effect of SF is more fully realized in the later stages.

The blank specimen (Test No. 0) is a Portland cement specimen, with an electric flux of 2095.3 C at 28 days. The electric flux of PCE-S1 is approximately 18.3% lower than that of the blank specimen, while PCE-W1 only shows a reduction of 4.6%. This difference can be attributed to the relatively higher air-entraining content in the liquid superplasticizer, which increases the number of pore structures within the cementitious matrix, thereby reducing its ability to resist chloride ion penetration. In the PCE-S8 and PCE-W8 mix designs, both contain a high dosage of mineral admixtures. The focus is on the change in electric flux at 56 days, where PCE-S8 exhibits a stronger ability to resist chloride ion penetration. In engineering applications, it is often necessary to combine it with other powder-based admixtures to improve the workability of the mixture during construction. Therefore, PCE-S8, which incorporates powder-based superplasticizers, should be prioritized as the benchmark mix.

#### 3.4.3. Experimental Study on Permeability Resistance

**Figure 22:**
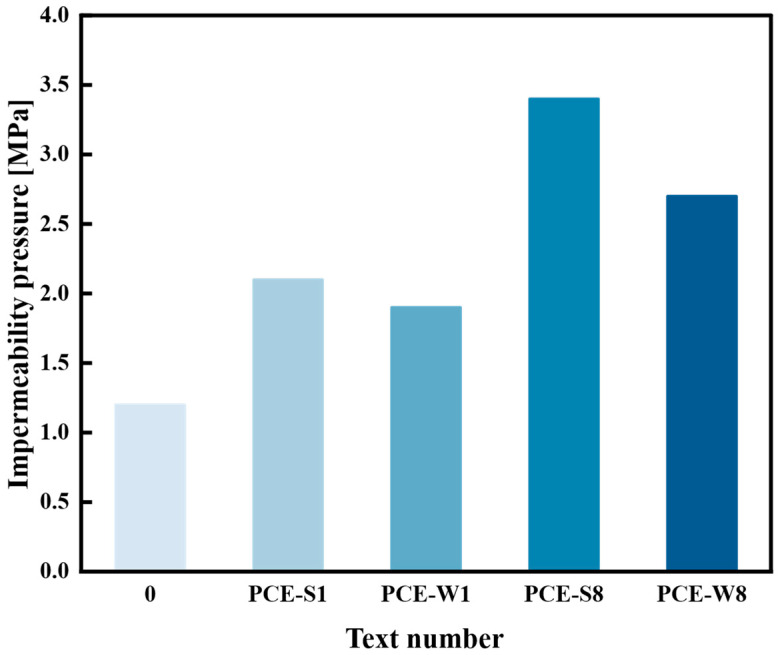
Water permeability pressure of each mix design.

The permeability test was conducted on four composite cementitious mortars, namely PCE-S1, PCE-W1, PCE-S8, and PCE-W8. The initial water pressure was set to 0.2 MPa; the maximum water pressure was set to 4.2 MPa. During the test, the permeability device automatically increased the water pressure by 0.1 MPa every hour. Throughout the test, the water pressure was observed and recorded every 0.5 h. The calculation followed Equation (2). The test used specimen No. 0 as the control and the maximum water penetration pressure values for all five groups, including the control, were obtained. The results are shown in Figure 22.

As shown in Figure 22, the incorporation of high-performance superplasticizers can improve the water permeability resistance of composite cementitious mortars. The water permeability pressure of PCE-S1 and PCE-W1 increased by 58.3% and 75.0%, respectively, compared to the blank specimen (Test No. 0) without superplasticizers. This is likely because the negative ions in the superplasticizer are adsorbed by the positively charged cement particles, causing electrostatic repulsion that leads to particle dispersion and water release. This improves the workability while reducing the porosity of the cement-based material and enhancing its water permeability resistance. Additionally, the cementitious mortar with liquid superplasticizer has a higher pore structure quantity than the one with solid superplasticizer, which results in lower water permeability resistance. Specifically, the water permeability pressure of PCE-W1 is 1.9 MPa, which is lower than the 2.1 MPa of PCE-S1.

The incorporation of mineral admixtures, in addition to superplasticizers, significantly enhances the water permeability resistance of composite cementitious mortars. For the PCE-W8 mix, the water permeability pressure increased by 42.1% compared to PCE-W1. The improvement is even more pronounced with the use of a solid superplasticizer, where PCE-S8 exhibited a 61.9% increase in water permeability pressure relative to PCE-S1. These findings indicate that the combined application of superplasticizers and mineral admixtures is highly effective in improving the impermeability performance of composite cementitious mortars.

At early ages, the partial replacement of cement reduces the amount of clinker available for hydration, thereby lowering the initial exothermic reaction. As hydration proceeds, the pozzolanic reaction between the amorphous silica and alumina phases in FA and GGBS with calcium hydroxide (CH) leads to the delayed formation of additional C–S–H gel. This secondary hydration process not only refines the pore structure but also contributes to long-term strength development. Furthermore, the fine particles of silica fume act as microfillers, occupying voids within the cement matrix, enhancing particle packing density, and reducing capillary porosity. This filler effect, combined with the ongoing pozzolanic reaction, results in a denser microstructure with fewer interconnected pores, as confirmed by the SEM observations. The synergistic contribution of these mechanisms explains the reduced total porosity and improved durability properties observed in the composite system.

### 3.5. Microstructural Analysis of Marine Engineering High-Performance Composite Cementitious Mortars

The hydration products of the different mix designs were analyzed and their morphology was observed using techniques such as X-ray diffraction (XRD) and scanning electron microscopy (SEM). The specimens selected for analysis included the blank specimen (Test No. 0), the mix design with solid superplasticizer (PCE-S1), and the mix design with both superplasticizer and mineral admixtures (PCE-S8). The hydration ages for the 0 and PCE-S1 mix designs were 3 d, 7 d, 28 d, and 56 d, while the hydration ages for the PCE-S8 mix design were 6 h, 24 h, 3 d, 7 d, 28 d, and 56 d.

#### 3.5.1. XRD Analysis

The phase characterization analysis was conducted using a Bruker D8 Advance X-ray Powder Diffraction (XRD) instrument (Bruker Corporation, Billerica, MA, USA). For the XRD analysis, the scanning parameters, including the scanning angle range, step size, and scanning speed, were set as follows: the scanning range was 10° to 70°, the step size was 0.02°, and the dwell time was 0.01 s.

**Figure 23:**
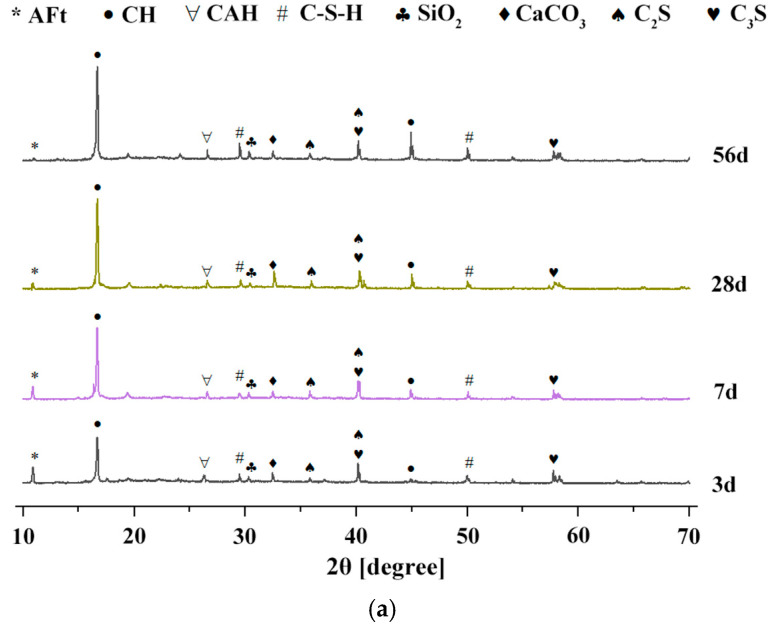

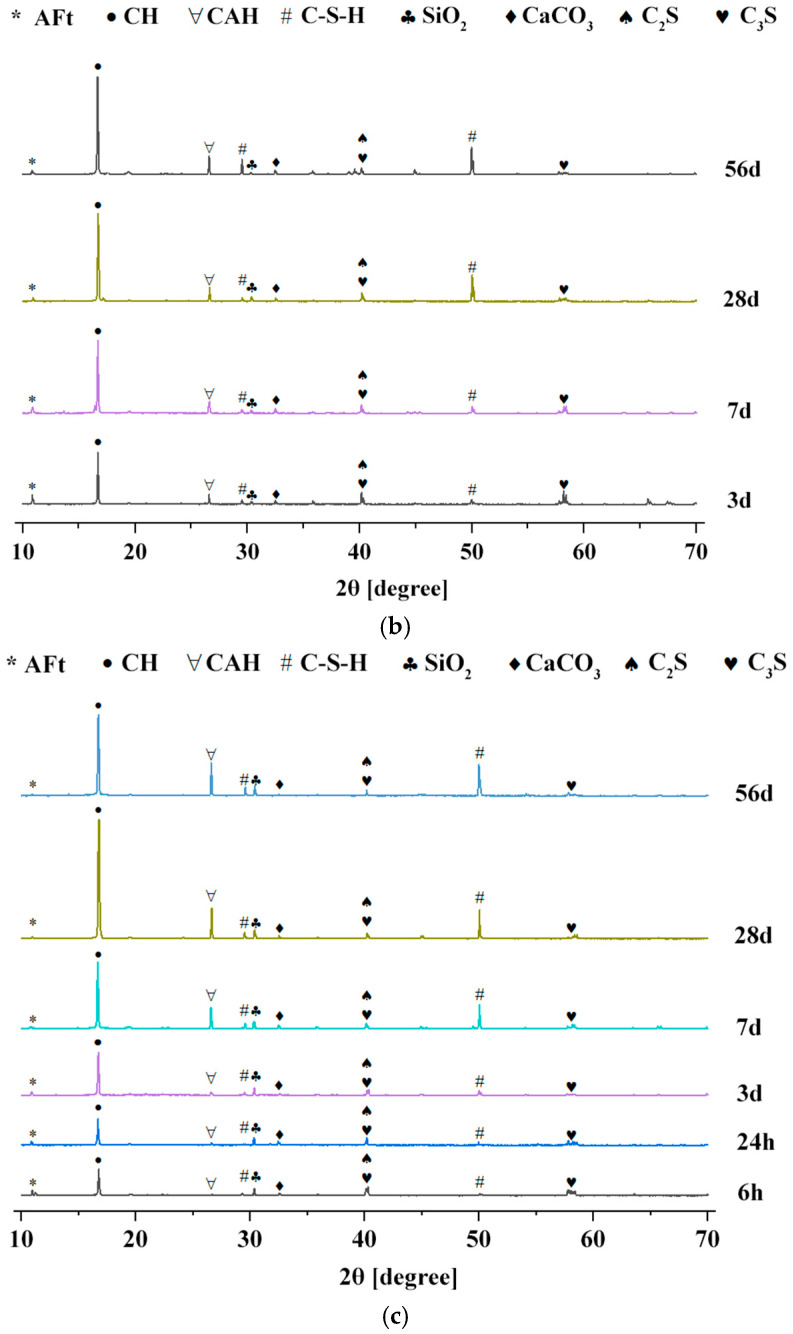
XRD patterns of different mix designs at different curing ages: (**a**) XRD patterns of 0 mix design at different curing ages; (**b**) XRD patterns of PCE-S1 mix design at different curing ages; and (**c**) XRD patterns of PCE-S8 mix design at different curing ages.

Figure 23 shows the X-ray diffraction patterns of three different mix designs of composite cementitious pastes at different ages. The horizontal axis represents the X-ray scanning angle, and the vertical axis represents the peak intensity.

As shown in Figure 23a, for the 0 mix design, the main hydration products at early curing ages are CH and several unhydrated cement minerals, such as C_3_S and C_2_S, along with a small amount of ettringite (AFt). As hydration progresses, the diffraction peaks corresponding to the cement minerals gradually decrease. However, even after 28 days, small amounts of unhydrated minerals remain, while the diffraction peaks of CH and other hydration products tend to stabilize, exhibiting minimal changes in intensity. As shown in Figure 23b, in the PCE-S1 mix design, the cement mineral phases C_3_A and C_4_AF begin to hydrate, while the relatively abundant C_3_S also accelerates its hydration reaction. During this process, the hydration of C_3_S generates CH crystals and C–S–H gel, while the hydration product of C_3_A is calcium aluminate hydrate (CAH) crystals. This indicates that the addition of polycarboxylate-based high-performance superplasticizer can enhance the early hydration rate of cement, improving its early strength. Furthermore, in the later stages of hydration, components such as C_3_S and C_3_A continue to hydrate, generating more hydration products, like CH and CAH, which contribute to improving the mechanical properties.

Compared to the PCE-S1 mix design without mineral admixtures, the XRD spectrum of the PCE-S8 mix shows a significant decrease in the intensity of the diffraction peaks of C_3_S and C_2_S, as shown in Figure 23c. This reduction is mainly due to the decrease in cement content, which directly leads to the reduction in C_3_S and C_3_A. As a result, the generation of corresponding hydration products also decreases, which is reflected in the weaker CH diffraction peak generated during the early hydration reaction. Additionally, it indicates that the active components of the mineral admixtures promote the hydration of C_3_S and reduce the content of CH. By 28 days, the diffraction peak of CH crystals noticeably increases, while the content of cement mineral phases, such as C_3_S, continues to decrease. A large amount of C–S–H gel, CAH, and other hydration products are generated and their peak intensity is significantly enhanced.

At 56 days, the CH content shows a decreasing trend, while the other hydration products increase. During the early hydration process, the rate at which mineral admixtures consume CH is lower than the rate at which CH is generated by cement hydration. Around 28 days, the consumption rate and generation rate are approximately balanced, and the CH content remains relatively stable. As the curing period continues, the consumption rate of CH gradually exceeds the generation rate, leading to a decrease in CH content at 56 days. After the incorporation of mineral admixtures, such as fly ash and silica fume, the composite cementitious materials contain a certain amount of silicon dioxide (SiO_2_). As a result, the X-ray diffraction patterns show a diffraction peak for SiO_2_, with a stronger intensity compared to the patterns of mixtures without mineral admixtures. As seen in Figure 23c, the peak intensity of C–S–H in the early hydration phase changes only slightly with age. This is primarily because the reactivity of mineral admixtures is not fully activated in the early stages and their hydration degree and rate are relatively low.

#### 3.5.2. SEM Analysis

**Figure 24:**
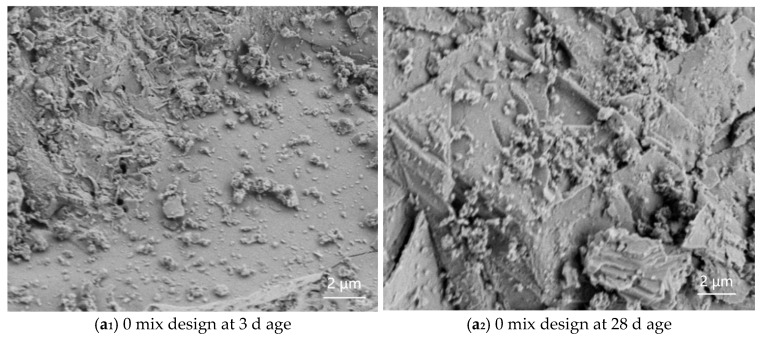

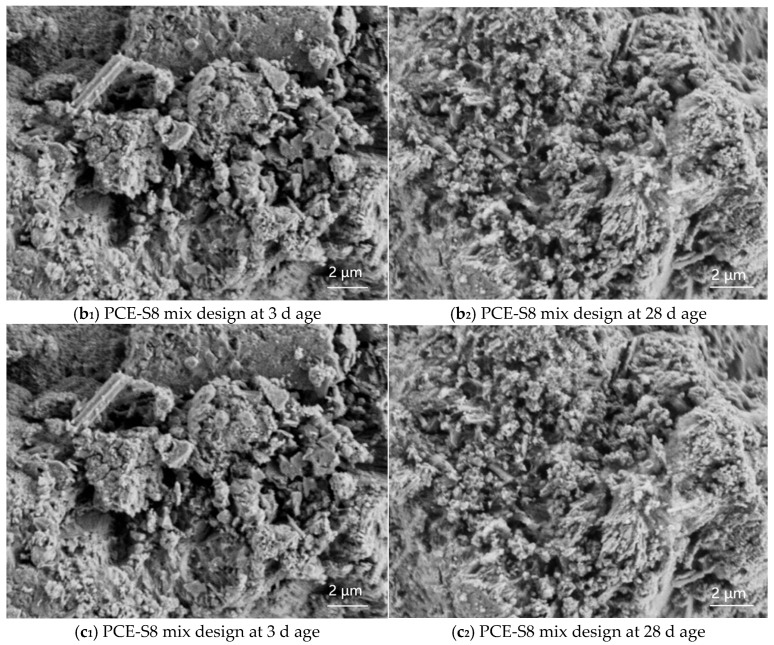
SEM images of composite cementitious materials for each mix design.

The experiment used a Zeiss Sigma-500 field emission scanning electron microscope (SEM, Carl Zeiss AG, Oberkochen, Germany) to observe the microstructure of four different mix designs of composite cementitious pastes at 3 d and 28 d curing ages, as shown in Figure 24. The images were magnified 10,000 times, with a scanning voltage of 2 kV and a working distance of 6.0 mm.

As shown in Figure 24(a_1_,a_2_), at the 3-day curing age of the 0 mix design, a large amount of gel-like cement minerals, such as C_3_S, can be clearly observed. At the 28-day curing age, hexagonal plate-like hydration products of CH crystals are visible, as well as needle-like AFt crystals, which are insoluble in water. Since the formation of AFt is relatively slow, it crystallizes on the surface of cement particles, hindering their rapid hydration. As the curing age increases, the overall microstructure tends to become denser and the pore area continuously decreases. As shown in Figure 24(b_1_,b_2_), compared to the 0 mix design, the PCE-S1 mix incorporating a superplasticizer exhibits a significant reduction in unhydrated cement mineral phases. A large quantity of fibrous C–S–H and hexagonal plate-like CH crystals is observed, filling the voids previously occupied by cement particles and water. The composition and morphology of C–S–H are similar to those of hydrated C_3_S, both being gel-like materials with variable compositions and low degrees of crystallinity, resulting in relatively poor compactness.

In Figure 24(c_1_,c_2_), it can be seen that the PCE-S8 mix shows little reaction at 3 days; however, at 28 days, a distinct gel-like flocculent structure is formed. This indicates that the hydration products of the mineral admixtures are similar to those of Portland cement, with both generating C–S–H gel, with differences in the contents of Ca, Si, Al, and other elements within the gel. Additionally, the calcium aluminate further hydrates, primarily forming plate-like crystalline hydration products of CAH, with a small portion transforming into plate-like hydrated calcium sulfoaluminate (AFm). This reaction occurs rapidly, forming a loose network structure that facilitates the rapid setting of the cement paste.

## 4. Conclusions

This study employed a dual-admixture experimental design along with controlled single-factor testing to preliminarily investigate the effects of superplasticizer type, mineral admixture dosage, and internal incorporation ratio on the main performance indicators of marine engineering high-performance composite cementitious mortars. Based on these results, the optimal mix design was determined. Using this as a foundation, performance research and microscopic analysis were conducted on different mix designs of marine engineering high-performance composite cementitious mortars. The main conclusions are as follows:

1. Optimal mix design and performance.
  The optimal mix design was determined as a water-to-binder ratio of 0.28, a solid PCE dosage of 0.95%, and a total mineral admixture content of 40% with an FA: GGBS:SF ratio of 1:3:0.6. This formulation achieved enhanced workability, mechanical strength, durability, and volume stability. Notably, the compressive strength reached 75.7 MPa at 56 days, while the bond strength exhibited a 46.2% improvement over that of ordinary Portland cement mortars;
2. Durability under simulated marine exposure.
  The optimized composite mortar successfully withstood more than 200 freeze–thaw cycles with strength loss and mass loss within acceptable limits. Chloride ion electric flux decreased by 58.9% at 56 days, and impermeability pressure increased by 2.4 times compared to the control. These results confirm the material’s strong potential for applications in offshore platforms, underwater tunnel linings, and coastal defense structures such as breakwaters;
3. Microstructural improvement mechanisms.
  XRD and SEM analyses showed that the addition of mineral admixtures promotes secondary hydration and the formation of dense C–S–H gel while consuming CH. This leads to a more refined microstructure, improving overall compactness and long-term durability;
4. Practical Significance and Engineering Potential.
  The proposed composite system offers an effective material solution for durable and sustainable marine construction, especially for repair and strengthening of concrete structures exposed to aggressive marine environments;
5. Limitations and future work.
  This study was based on laboratory-scale tests under simulated conditions. Long-term performance in real marine environments, cyclic fatigue under wave loads, and adhesion behavior with embedded steel reinforcement require further investigation. Future work should include field validation, rheological optimization during construction, and a life-cycle environmental impact assessment to support practical engineering implementation.

## Figures and Tables

**Table 1:** Chemical composition of raw materials.

Material	CaO	SiO_2_	Al_2_O_3_	Fe_2_O_3_	MgO	SO_3_	LOI
Cement	58.44	23.27	5.83	4.01	4.46	2.39	4.77
FA	2.15	53.12	29.98	6.34	0.55	1.70	2.20
GGBS	34.00	34.50	17.70	1.03	6.01	1.64	0.84
SF	0.23	97.35	0.21	1.09	0.39	0.73	1.63

**Table 2:** Basic performance characteristics of superplasticizers.

Specifications	Setting Time Interval [min]		Compressive Strength Ratio [%]				Water Reduction Rate [%]	Air Content [%]	Bleeding Rate Ratio [%]
	Initial Setting Time	Final Setting Time	1 d	3 d	7 d	28 d			
PCE-S	15	20	195	186	172	152	40	2.8	22
PCE-W	30	25	175	165	160	/	40	4.0	13

**Table 3:** Optimal mix designs for plan 1 and plan 2.

Test No.	Superplasticizer Dosage [%]	Water Dosage [g]	Water-to-Binder Ratio
PCE-S1	0.95	130	0.28
PCE-W1	1.0	128	0.33

**Table 4:** Scheme 3 (1) mix design and results of composite cementitious mortar (FA:GBSS = 1:3).

Test No.	Cement Content [%]	Total Content of FA and GBSS [%]	Flowability [mm]	Flexural Strength [MPa]		Compressive Strength [MPa]	
				28 d	56 d	28 d	56 d
PCE-S1	100	0	159	15.1	15.4	62.3	65.6
PCE-S2	90	10	150	15.9	16.4	64.6	68.4
PCE-S3	80	20	150	16.0	16.6	66.9	69.4
PCE-S4	70	30	152	16.7	17.3	68.4	70.7
PCE-S5	60	40	156	16.9	17.9	70.4	72.1
PCE-S6	50	50	166	16.1	17.1	68.9	69.2

**Table 5:** Scheme 3 (2) mix design and results of composite cementitious mortar (FA:GBSS:SF = 1:3:X).

Test No.	Cement Content [%]	Total Dosage of SCM [%]	Three SCM Ratios: FA:GBSS:SF	Flowability [mm]	Flexural Strength [MPa]		Compressive Strength [MPa]	
					28 d	56 d	28 d	56 d
PCE-S7	60	40	1:3:0.2	171	17.1	18.3	70.2	75.3
PCE-S8	60	40	1:3:0.6	178	17.5	18.9	73.1	75.7
PCE-S9	60	40	1:3:1.0	194	17.0	17.6	69.7	73.6

**Table 6:** Scheme 4 (1) mix design and results of composite cementitious mortar (FA:GBSS = 1:3).

Test No.	Cement Content [%]	Total Content of FA and GBSS [%]	Flowability [mm]	Flexural Strength [MPa]		Compressive Strength [MPa]	
				28 d	56 d	28 d	56 d
PCE-W1	100	0	157	15.4	15.7	61.5	62.3
PCE-W2	90	10	145	16.3	16.9	66.3	67.0
PCE-W3	80	20	150	15.8	16.2	63.0	65.7
PCE-W4	70	30	154	14.8	16.4	61.4	67.2
PCE-W5	60	40	170	16.3	17.2	65.4	67.4
PCE-W6	50	50	168	15.9	16.4	63.4	67.0

**Table 7:** Scheme 4 (2) mix design and results of composite cementitious mortar (FA:GBSS:SF = 1:3:X).

Test No.	Cement Content [%]	Total Dosage of SCM [%]	Three SCM Ratios: FA:GBSS:SA	Flowability [mm]	Flexural Strength [MPa]		Compressive Strength [MPa]	
					28 d	56 d	28 d	56 d
PCE-W7	60	40	1:3:0.2	151	16.1	17.2	65.8	70.3
PCE-W8	60	40	1:3:0.6	156	16.7	18.1	70.3	72.6
PCE-W9	60	40	1:3:1.0	160	16.5	17.5	68.6	72.1

**Table 8:** Mass loss rate of each mix design in freeze–thaw cycles (unit: %).

	Test No.	0	PCE-S1	PCE-W1	PCE-S8	PCE-W8
Cycles						
25		−0.06	−0.08	−0.09	−0.02	−0.05
50		0.82	0.59	0.74	0.52	0.68
75		1.68	0.95	1.33	0.89	1.21
100		3.55	1.76	2.06	1.74	1.99
125		4.45	2.26	2.69	2.02	2.59
150		9.17	3.44	3.65	3.27	3.46
175		—	4.76	5.52	4.11	4.71
200		—	5.83	—	4.46	5.13

**Table 9:** Compressive strength loss rate of each nix design in freeze–thaw cycles (Unit: %).

	Test No.	0	PCE-S1	PCE-W1	PCE-S8	PCE-W8
Cycles						
25		2.42	1.38	2.30	−0.92	−1.24
50		4.38	3.85	4.11	2.54	3.67
75		9.17	7.64	8.37	6.24	7.35
100		15.06	12.60	13.35	8.02	9.42
125		24.64	17.58	18.10	10.62	11.38
150		39.36	24.47	27.01	14.18	15.79
175		—	32.95	—	18.91	21.71
200		—	—	—	21.83	24.20

## Data Availability

The original contributions presented in this study are included in the article. Further inquiries can be directed to the corresponding author.

## Abbreviations

The following abbreviations are used in this manuscript:

PCE	Polycarboxylate superplasticizer
PCE-S	Solid Polycarboxylate Superplasticizer
PCE-W	Liquid Polycarboxylate Superplasticizer
FA	Fly ash
GGBS	Ground granulated blast furnace slag
SF	Silica fume
XRD	X-ray diffraction
SEM	Scanning electron microscopy
C-S-H	Calcium silicate hydrate
SCM	Supplementary cementitious material
OPC	Ordinary Portland cement
CAH	Calcium sulfoaluminate hydrate
CH	Calcium hydroxide
AFt	Ettringite
SiO_2_	Silicon dioxide
AFm	Hydrated calcium sulfoaluminate
